# Alteration in brain-derived neurotrophic factor (BDNF) after treatment of mice with herbal mixture containing Euphoria longana, Houttuynia cordata and Dioscorea japonica

**DOI:** 10.1186/s40199-014-0077-2

**Published:** 2014-11-28

**Authors:** Songhee Jeon, Chia-Hung Lee, Quan Feng Liu, Geun Woo Kim, Byung-Soo Koo, Sok Cheon Pak

**Affiliations:** Dongguk University Research Institute of Biotechnology, Seoul, 100-715 Republic of Korea; Department of Neuropsychiatry, Graduate School of Oriental Medicine, Dongguk University, Gyeongju, Republic of Korea; Department of Korean Neuropsychiatry, Dongguk University Bundang Oriental Hospital, Sungnam, Republic of Korea; Department of Korean Neuropsychiatry, Dongguk University Ilsan Oriental Hospital, Goyang, Republic of Korea; School of Biomedical Sciences, Charles Sturt University, Bathurst, NSW 2795 Australia

**Keywords:** Stress, Depression, BDNF, Memory, *Euphoria longana*, *Houttuynia cordata*, *Dioscorea japonica*

## Abstract

**Background:**

Literature data indicate that brain-derived neurotrophic factor (BDNF), cyclic-AMP response element-binding protein (CREB) and phospho-CREB (pCREB) may have a place in depression. BDNF belongs to the neurotrophin family that plays an important role in proliferation, survival and differentiation of different cell populations in the mammalian nervous system. The herbal mixture used in the present study consists of *Euphoria longana*, *Houttuynia cordata* and *Dioscorea japonica*. The purpose of the present study was to determine the neuroprotective effect of herbal mixture. We also tested the hypothesis that administration of herbs reverses memory deficits and promotes the protein expression of BDNF in the mouse brain.

**Methods:**

Mice were randomized into four different treatment groups (n = 10/group). Normal and stress groups received regular lab chow without stress and under stress conditions, respectively, for 3 weeks. The animals in the stress group were immobilized for 4 hours a day for 2 weeks. Different doses of herbal mixture (206 and 618 mg/kg) were administered for 3 weeks to those mice under stress conditions. Mice were analyzed by behavioral tests and immunoblotting examination in the hippocampus and cortex. An additional in vitro investigation was performed to examine whether herbs induce neurotoxicity in a human neuroblastoma cell line, SH-SY5Y cells.

**Results:**

No significant toxicity of herbs on human neuroblastoma cells was observed. These herbs demonstrated an inductive effect on the expression of BDNF, pCREB and pAkt. For spatial working memory test, herbal mixture fed mice exhibited an increased level of spontaneous alternation (p < 0.01) compared to those in stress conditions. Moreover, herbal mixture produced highly significant (p < 0.01) reduction in the immobility time in the tail suspension test. Mice in the herbal mixture groups demonstrated lower serum corticosterone concentration than mice in the stress group (p < 0.05). Effects of the oral administration of herbal mixture on protein levels of BDNF in the hippocampi and cortices were significant.

**Conclusions:**

Our study showed that herbal mixture administration has antidepressant effects in mice. It is proposed that adverse events such as stress and depression can modulate the expression of molecular players of cellular plasticity in the brain.

## Background

Specific data from the Global Burden of Disease 2010 study confirmed that mental and substance use disorders account for 7.4% of disease burden worldwide which is greater than HIV/AIDS and tuberculosis (5.3%), diabetes (2%) or transport injuries (3.3%) [1]. Depression in particular is projected to become the leading cause of disability and the second leading contributor to the global burden of disease in approximately 10 years [2]. As a heterogeneous disorder with unclear etiology, depression includes disturbance of neurogenesis, genetic predisposition, deficiency of monoamines, hypercortisolemia, reduction of neurotrophins, inflammation and oxidative stress [3].

It has been proposed that brain-derived neurotrophic factor (BDNF), as one of neurotrophins, may be a key player in depression since it is involved in regulation of neuronal differentiation, survival, function and plasticity [4]. This neurotrophic hypothesis of depression claims that decreased levels of BDNF contribute to the hippocampal atrophy seen in depressed patients [5]. Depressive symptoms are also associated with decreased serum level of BDNF [6]. These evidences have already led to clinical trials of antidepressant medications for treating major depression, although there are some gaps in the clinical outcomes depending on the classes of psychotropic drugs and uncertainty about whether drugs that specifically target depressive symptoms are feasible and safe. The transcription of BDNF is dependent on cyclic-AMP response element-binding protein (CREB).

CREB is a transcriptional factor implicated in the control of adaptive neuronal responses. It has been found that calcium ions in neurons bind to a calcium response element within the BDNF gene which triggers phosphorylation of CREB and subsequent activation of BDNF transcription [7]. Depression is accompanied by downregulated and decreased phosphorylated CREB (pCREB). A growing number of clinical and experimental studies have demonstrated that structural and functional modifications of hippocampus from antidepressant treatments most likely require the transcription and protein expression of CREB, despite some inconsistencies in its mRNA and protein levels depending on the brain regions where such molecule is expressed [7]. CREB is regulated by several pathways including protein kinase B (PKB/Akt).

Antidepressant medications are effective at treating depression, but discontinuation rates are high because adverse effects are common [8]. For example, selective serotonin reuptake inhibitors (SSRIs) frequently cause gastrointestinal problems, insomnia, headache, anxiety and sexual dysfunction although they are the most commonly prescribed antidepressants [9]. Considering the side effects of synthetic antidepressants, natural plants can be alternative sources of new antidepressant drugs due to their more tolerable and less toxic properties. Therefore, the current study was performed as the progress toward finding reliable alternatives to depression.

Three different ingredients of traditional Korean medicine for depression were selected to observe the effects of them in cell culture and animal experiment. *Euphoria longana* Lam., especially a dried longan seed extract, contain high levels of antioxidant polyphenolic compounds [10]. Longan seed extracts are found to possess anticancer properties by inducing apoptosis [11]. *Houttuynia cordata* Thunb. is a single species of its genus and the whole plant of *H. cordata* is used for the treatment of diabetes [12]. Reported pharmacological activities of plant include anticancer [13], anti-inflammatory [13] and antioxidant [14]. The rhizome of *Dioscorea japonica* Thunb. has been used to strengthen stomach function, improve anorexia, eliminate diarrhea, dilute sputum, and moisturize skin in traditional Chinese medicine [15]. An ethanol extract of the rhizome of *D. japonica* showed considerable cytotoxic activity against some human tumor cell lines [16]. These three natural plants have been used as traditional herbal medicines in the treatment of neurological disorders in Korea. However, few studies have evaluated their antidepressant effects. The effects of these three herbs on BDNF, pCREB and pAkt are currently unknown. In this experiment, one human neuroblastoma cell line was used to investigate the neuroprotective effect of herbs. We also tested the hypothesis that administration of herbs reverses memory deficits and promotes the protein expression of BDNF in the mouse brain. To test the hypothesis, three different settings of neuronal cell culture, behaviour study and their brains have been used in the present study.

## Methods

### Preparation of herbal extracts

All medicinal herbs used were purchased from DY Herb (Seoul, Korea). Each herb was boiled in 30% ethanol mixed filter-purified drinking water for 2 h at 100°C in a reflux condenser. For the cellular experiment, aqueous extract of each herb was freeze-dried and the dried extract was reconstructed with distilled water and filtered through 0.2 μm filter (Millipore, MA, USA). In addition, the resulting dry powder of each herb was mixed with the same ratio and added into the feed in the animal studies.

### Cell culture

Human neuroblastoma SH-SY5Y cells were obtained from American Type Culture Collection (Rockville, MD, USA). Cells were maintained in Dulbecco’s modified Eagle’s minimum essential medium (DMEM, Invitrogen, Carlsbad, CA, USA) supplemented with 10% fetal bovine serum (FBS, Invitrogen, Carlsbad, CA, USA), 100 unit/ml penicillin and 100 μg/ml streptomycin (Gibco-BRL, Rockville, MD, USA) with further addition of trypsin-EDTA (Gibco-BRL, Rockville, MD, USA) in condition of 95% air and 5% CO_2_ at 37°C.

### Cell viability assay

Cell viability was determined by 3-(4,5-dimethylthizaol-2-yl)-2,5-diphenyltetrazolium bromide (MTT, Abcam, Cambridge, MA, USA) assay. SH-SY5Y cells were seeded in 12-well plates (Corning Incorporated, USA) at a density of 5 × 10^3^ cells/well, stabilized at 37°C for 16 h in DMEM medium supplemented with FBS and cultured with the indicated concentrations of herbs for 24 h. The medium was removed and the cells were incubated with 2 mg/ml of MTT solution. After incubation for 4 h at 37°C and 5% CO_2_, the supernatants were removed and dimethyl sulfoxide (DMSO, Sigma, USA) was added. The reactants were measured in terms of optical density (OD) at 580 nm with a microplate reader (UV max, Molecular Devices, USA). The optical densities were converted into percentages using the following formula: Cell viability (%) = OD sample/OD negative control × 100. Negative control cells were treated with complete DMEM alone.

### Quantification of BDNF/pCREB/pAkt expression by Western blot analysis

SH-SY5Y cells were maintained in serum-free medium with herbs at 30 μg/ml for 24 h. Cells were then washed twice with ice-cold PBS and lysed in lysis buffer containing 62.5 mmol/L Tris–HCl, pH 6.8, 2% SDS, 20% glycerol, 10% 2-mercaptoethanol and protease inhibitors. After incubation for 30 min on ice, cell lysates were centrifuged and protein concentrations were determined using bicinchoninic acid method (Pierce). Samples of cell lysate containing 50 μg of total protein were separated by 4-12% SDS-PAGE and transferred onto nitrocellulose membrane (Amersham Pharmacia Biotech, Buckinghamshire, UK) by electroblotting. After blocking with 5% skim milk in Tris-buffered saline (50 mmol/L Tris–HCl, pH 7.6, 150 mmul/L NaCl, 0.1% Tween-20) for more than 30 min, the membranes were incubated for 16 h at 4°C with primary antibodies (anti-BDNF, anti-phosphorylated-CREB, anti-phosphorylated-Akt, β-actin antibody) and further incubated with horseradish peroxidase-conjugated secondary antibodies for 1 h. The membranes were visualized by an enhanced chemiluminescence system (ECL kit; Pierce, Rockford, IL, USA). Densitometric analysis was performed by Quantity One (Bio-Rad, Hercules, CA) to scan the signals. Further assay was carried out to detect BDNF protein expression in the hippocampus and cortex. The treated hippocampal and cortical tissues removed and homogenized in lysis buffer containing 50 mM Tris-base (pH 7.5), 150 mM NaCl, 2 mM EDTA, 1% glycerol, 10 mM NaF, 10 mM Na-pyrophosphate, 1% NP-40 and protease inhibitors (0.1 mM phenylmethylsulfonylfluoride, 5 μg/ml aprotinin, and 5 μg/ml leupeptin). Thirty μg of tissue lysate were electrophoresed using SDS-polyacrylamide gels and transferred to nitrocellulose membranes, which were then incubated with anti-BDNF (Cell signaling technology, Beverly, MA, USA) for 16 h at 4°C. After washing with TBS-T (0.05%), the blots were incubated with horseradish peroxidase-conjugated anti-rabbit or anti-mouse IgG, and the bands were visualized using the ECL system (Thermo Fisher Scienctific, USA). Band images were obtained by using a Molecular Imager ChemiDoc XRS^+^ (Bio-Rad, Hercules, CA, USA) and band intensity was analyzed using Image Lab™ software version 2.0.1 (Bio-Rad).

### Experimental animals

Protocols for animal use were reviewed and approved by the Institutional Animal Care and Use Committee at the Dongguk University Ilsan Hospital and were in accordance with National Institute of Health guidelines. Healthy male ICR mice (20–26 g, 6 weeks old) were obtained from OrientBio (Seoul, Korea) and were allowed 1 week for quarantine and acclimatization. Animals were housed under the conditions of constant temperature (22 ± 1°C), relative humidity (55 ± 1%) and 12 h light/12 h dark cycle (light on at 7:00 am). They were housed in polycarbonate cages and given tap water and commercial rodent chow (Samyang Feed, Daejeon, Korea) ad libitum. Forty mice were blindly randomized into four different treatment groups (n = 10/group). Normal and stress groups received regular lab chow without stress and under stress conditions, respectively, for 3 weeks. The animals in the stress group were immobilized for 4 h a day for 2 weeks by being gently inserted into a flexible triangle shaped vinyl screen that was closed and secured with nonallergic adhesive tape. Different doses of herbal mixture (206 and 618 mg/kg which are equivalent to the recommended adult human dose of 1000 and 3000 mg/kg, respectively) were administered for 3 weeks to those mice under stress conditions. The herbal mixture of *E. longana*, *H. cordata* and *D. japonica* was in a ratio of 1:1:1 by weight. Lab chow with or without herbal mixture was prepared for a mouse to have 1 g/kg/day which is equivalent to 7 g/60 kg/day for human and was gamma-irradiated. In order to examine the nutritional status, body weight was evaluated in mice that were fed with normal or herb mixture diet. At the end of experiment period, all animals were immediately decapitated, and their hippocampus and cortex of brains were collected and frozen at −80°C for following analysis. Blood was collected from the heart by cardiac puncture prior to the excision of brain, then after centrifugation serum aliquots were stored at −70°C for further corticosterone determination. The concentration of serum corticosterone was measured with the Corticosterone EIA Kit (Enzo Life Sciences, Farmingdale, NY, USA) in accordance with the manufacturer’s instructions.

### Step-through passive avoidance test

The apparatus (AP model; O’Hara Co., Tokyo, Japan) for the step-through passive avoidance test consisted of two compartments, an illuminated compartment [100 mm · 120 mm · 100 mm; light at the top of compartment (27 W, 3000 lx)] and a dark compartment (100 mm · 170 mm · 100 mm). The compartments were separated by a guillotine door. During the training trial, a mouse was placed in the safe, illuminated compartment. As the compartment was lit, the mouse stepped through the opened guillotine door into the dark compartment. The time spent in the illuminated compartment was defined as the latency time. Three seconds after the mouse entered the dark compartment, a foot shock (0.3 mA, 50 V, 50 Hz ac for 3 seconds) was delivered to the floor grids in the dark compartment. The mouse could escape from the shock only by stepping back to the safe illuminated compartment. Mice remaining in the light chamber for more than 120 seconds during the learning stage were excluded from the following retention trial. The retention trials were carried out at 24 h after the training trial to evaluate the retention of avoidance memory. The latency time was measured for up to 300 seconds without delivering a foot shock. It was judged that the mouse retained the avoidance memory when it stayed in the illuminated safe compartment for 300 seconds.

### Y-maze test

This behavioral test was performed at 48 h after the passive avoidance test. The maze was made of black painted wood; each arm was 40 cm long, 12 cm high, 3 cm wide at the bottom and 10 cm wide at the top. The arms converged at an equilateral triangular central area that was 4 cm at its longest axis. Each mouse was placed at the centre of the apparatus and allowed to move freely through the maze for 8 min. The series of arm entries was recorded visually. Alternation was defined as successive entry into the three arms on overlapping triplet sets. Alternation behavior (%) was calculated as the ratio of actual alternations to possible alternations (defined as the number of arm entries minus two) multiplied by 100.

### Forced swimming test

Mice were placed in a Plexiglas cylinder 25 cm in height with a 15 cm internal diameter containing water at a temperature of 25 ± 1°C and a depth of 10 cm so they could not escape and could not touch the bottom. Water was changed between each swim session to prevent possible effects of an alarm substance released by mice during the swim session. There were two swim sessions. The first was 15 min pre-test swim for 3 days in a row and a second 5 min swim test. The pre-test facilitates the development of immobility during the test session and increases the sensitivity for detecting antidepressant behavioral effects. The 5 min swim test was used for analysis of behavior such as swimming, climbing and immobility.

### Tail suspension test

Mice both acoustically and visually isolated were suspended individually by their tails 40 cm above the tabletop with the use of an adhesive tape placed approximately 1 cm from the tip of the tail. After a few minutes of vigorous activity, the mice hung passively and completely motionless. The total immobility period in number of seconds was scored throughout the 6 min test. Mice were considered immobile with the absence of any limb or body movements, except for those caused by respiration. A decrease in the duration of immobility is indicative of an antidepressant-like effect [17].

### Data analysis

All statistical analyses were conducted with SPSS (ver. 19, Somers, NY, USA). Values are expressed as means ± SEM. All data were analyzed using Student’s *t*-test. Statistical significance was accepted at a *p* value less than 0.05.

## Results

### Effect of herbs on cell viability

**Figure 1:**
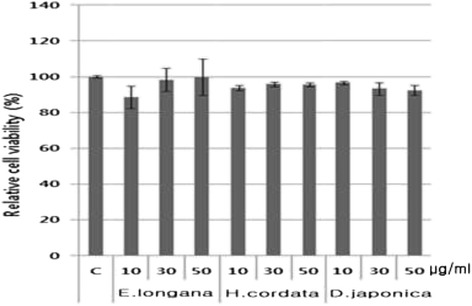
**Effect of herbs on the cell viability measured by MTT assay.** The human neuroblastoma SH-SY5Y cells were incubated at concentrations of 10, 30 and 50 μg/ml of each herb for 24 h. Values are presented as means ± SEM of six determinations.

In order to measure the cytotoxicity of herbs on SH-SY5Y neuroblastoma cells, a cell viability using MTT assay was utilized. Cells were treated with herbs at concentrations of 10, 30 and 50 μg/ml for 24 h (Figure 1). The percentages of viable cells were as follows: *E. longana* 10 μg/ml, 88.53 ± 6.3%; 30 μg/ml, 98.25 ± 6.4%; 50 μg/ml, 99.73 ± 10.1%; *H. cordata* 10 μg/ml, 93.76 ± 1.6%; 30 μg/ml, 95.83 ± 1.2%; 50 μg/ml, 95.61 ± 1.0%; and *D. japonica* 10 μg/ml, 96.58 ± 0.9%; 30 μg/ml, 93.22 ± 3.4%; 50 μg/ml, 92.38 ± 2.8%. No significant toxicity of herbs on SH-SY5Y was observed at the indicated concentrations.

### Effect of herbs on BDNF/pCREB/pAkt expression

**Figure 2:**
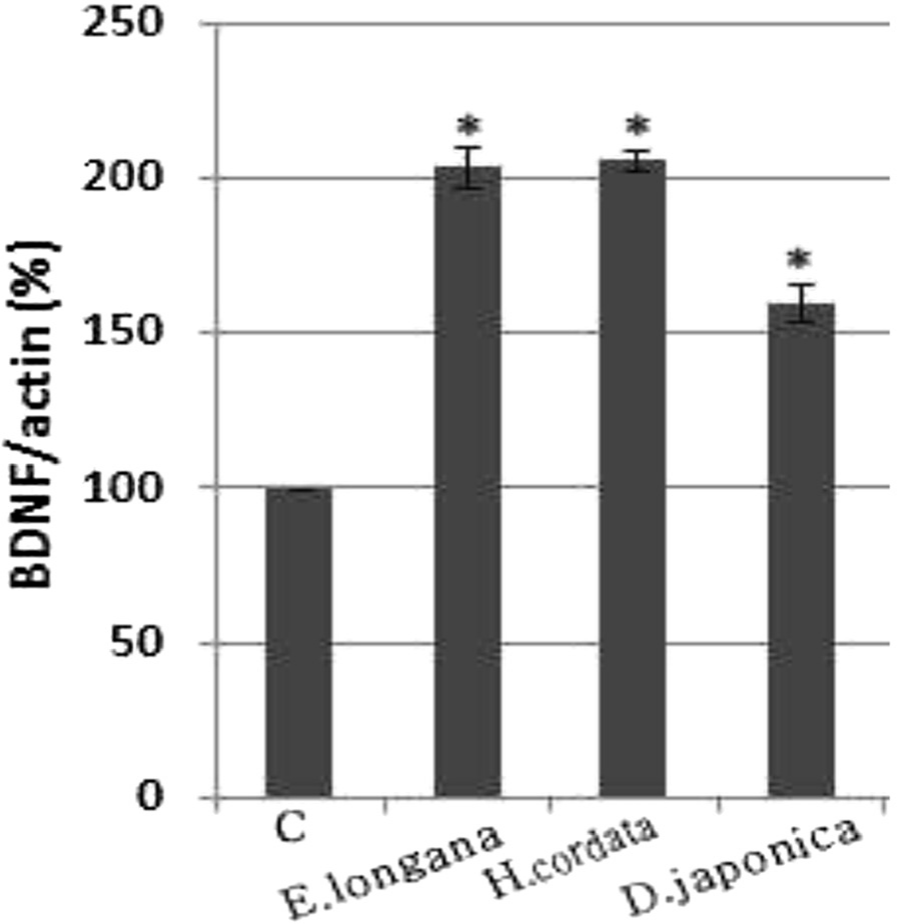
**Measurement of BDNF level in neuroblastoma SH-SY5Y cells.** To evaluate the protein level of BDNF expression, cells were incubated with 30 μg/ml herb. For the control, nonincubated cells were analyzed. The BDNF level in cell lysates was measured by immunoblotting. Data are presented as means ± SEM of three determinations. ^*^ *P* < 0.05 vs. control.

**Figure 3:**
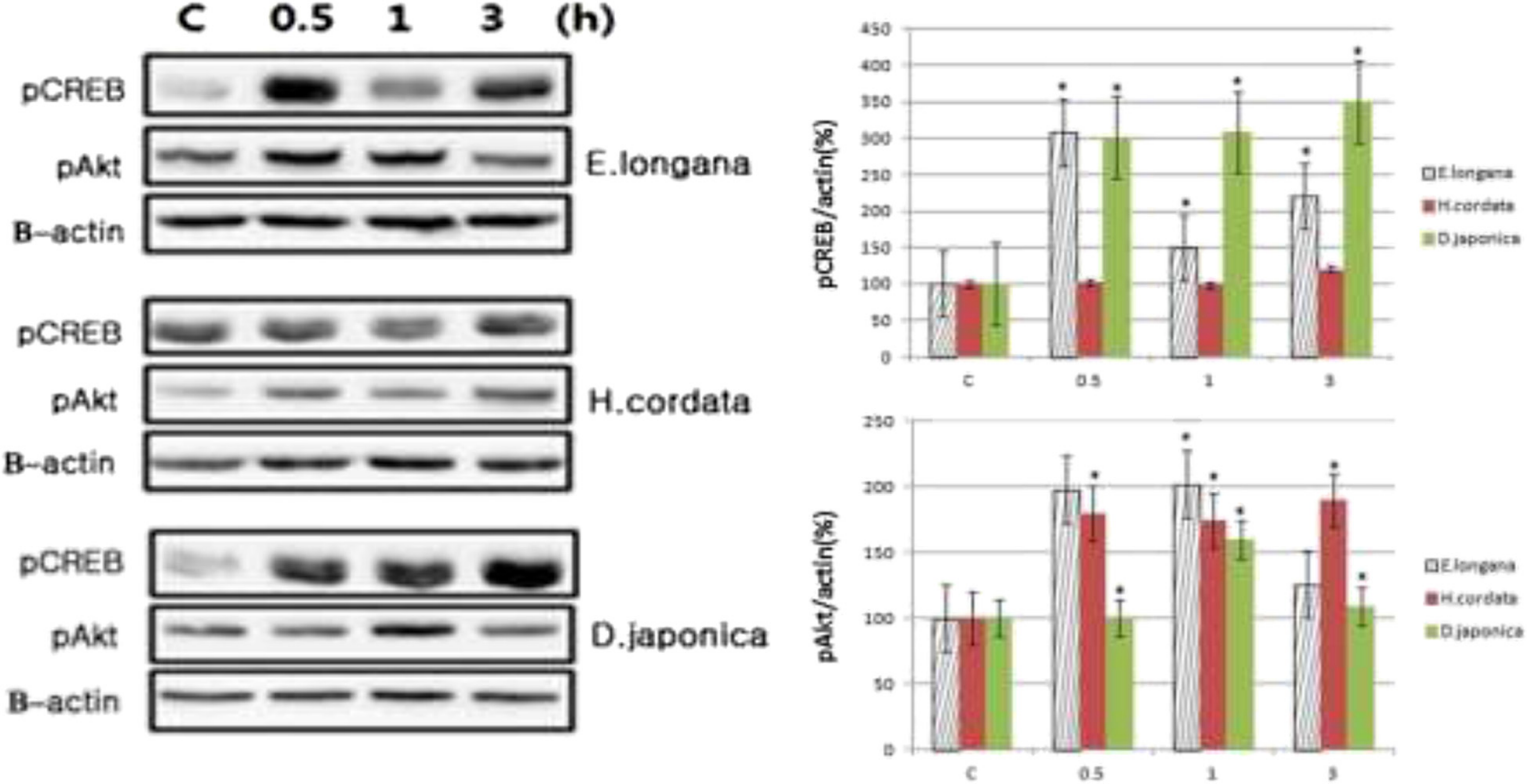
**Time course of expression of pCREB and pAkt in neuroblastoma SH-SY5Y cells.** To evaluate the protein level of pCREB and pAkt expression, cells were incubated with 30 μg/ml herb. For the control, nonincubated cells were analyzed. The level of pCREB and pAkt in cell lysates was measured by immunoblotting. Data are presented as means ± SEM of three determinations. ^*^ *P* < 0.05 vs. control.

We looked at the inductive effects of herbs on the expression of BDNF, pCREB and pAkt in cell extracts. For the control, nonincubated SH-SY5Y cells were analyzed. The BDNF expression in the SH-SY5Y cell extracts treated with herbs was significantly higher than that in the control cells (Figure 2). Immunoblotting analysis confirmed these results (figure not shown). To determine the expression of pCREB and pAkt, the cultures were harvested at 30 min, 1 h and 3 h and immunoblotting was performed on the cell extracts (Figure 3). Compared to the control, the expression of pCREB treated with 30 μg/ml of *E. longana* was 308 ± 8%, 150 ± 11% and 220 ± 10% at 30 min, 1 h and 3 h, respectively. For *H. cordata*, it was 102 ± 11%, 98 ± 15% and 120 ± 12% at 30 min, 1 h and 3 h, respectively. In the case of *D. japonica*, it was 302 ± 11%, 308 ± 15% and 350 ± 14% at 30 min, 1 h and 3 h, respectively. The pAkt expression in the cell extracts that were incubated with 30 μg/ml of *E. longana* was 198 ± 11%, 202 ± 15% and 126 ± 16% at 30 min, 1 h and 3 h, respectively. For *H. cordata*, it was 180 ± 11%, 175 ± 10% and 190 ± 9% at 30 min, 1 h and 3 h, respectively. In the case of *D. japonica*, it was 100 ± 10%, 160 ± 12% and 109 ± 17% at 30 min, 1 h and 3 h, respectively. Overall each herb was able to induce the expression of pCREB and pAkt regardless of time course.

### Antidepressant effect of herbal mixture

**Figure 4:**
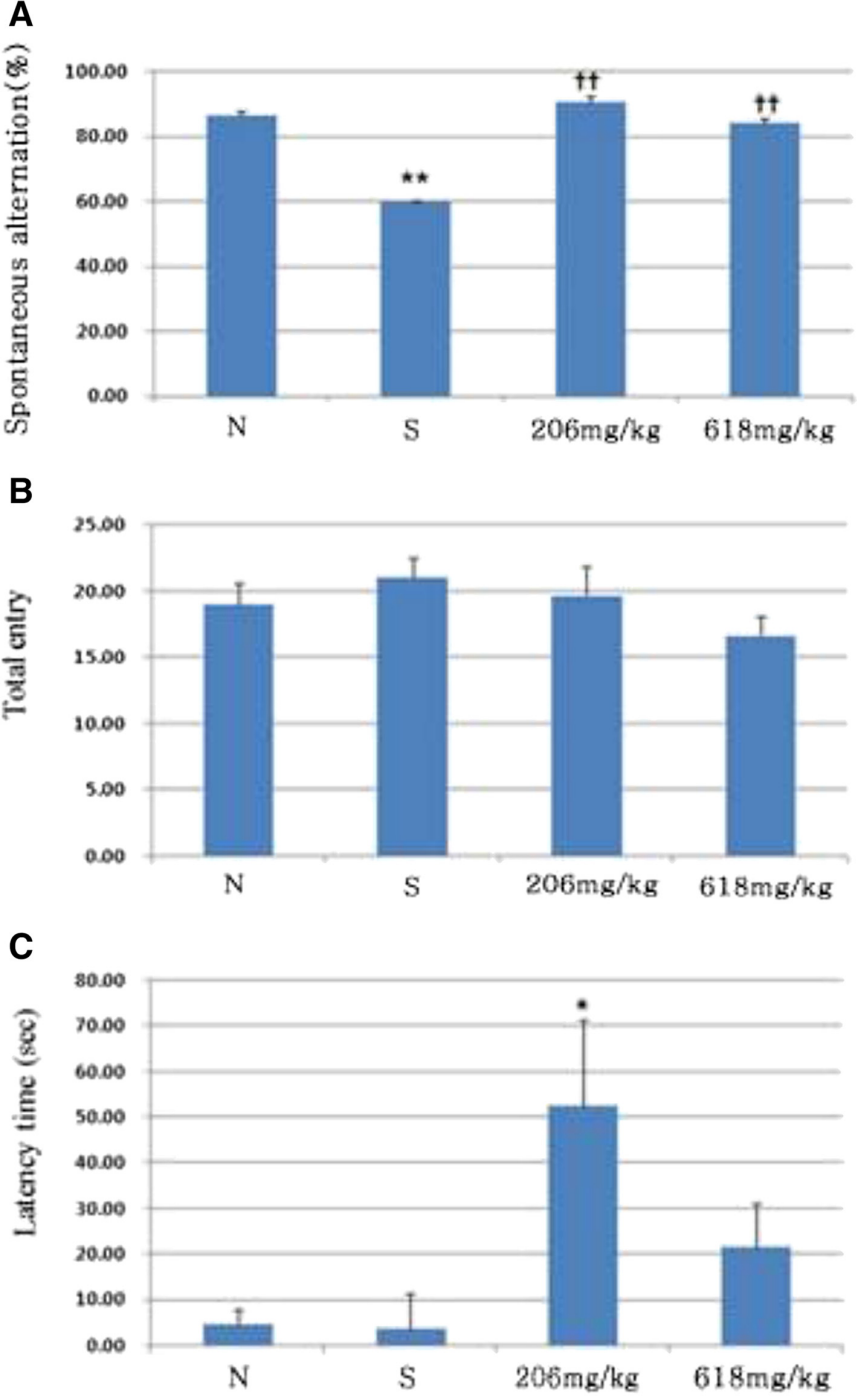
**Attenuated effect of herb mixture on stress induced impairment of the Y-maze task and passive avoidance task in mice.** Spontaneous alternation in behavior **(A)** and the number of arm entries **(B)** were measured. **(C)** Mice were subjected to the training trial and the step-through latency time was measured. Data are presented as means ± SEM of three determinations. ^*^ *P* < 0.05 vs. stress, ^**^ *P* < 0.01 vs. normal, ^††^ *P* < 0.01 vs. normal.

**Figure 5:**
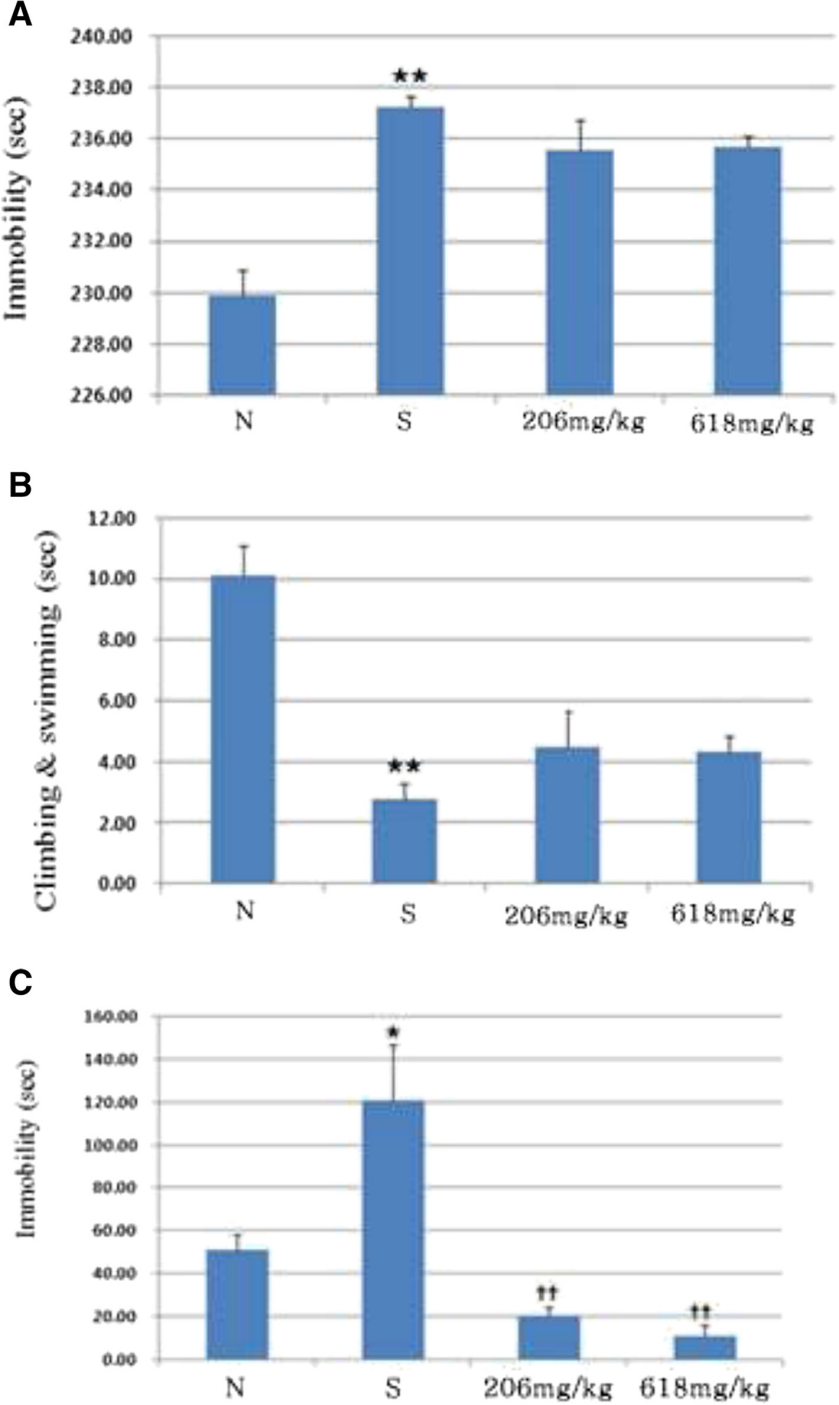
**Effect of herbal mixture on duration of immobility (A), swimming and climbing (B) behaviors in mice when sampled during the 5 min swim test. (C) Effect of herbal mixture on the amount of immobility in mice during the tail suspension test. Behavior was observed during the 6 min test period and scored as mobile or immobile.** Data are presented as means ± SEM of three determinations. ^*^ *P* < 0.05 vs. normal, ^**^ *P* < .01 vs. normal, ^††^ *P* < 0.01 vs. stress.

**Figure 6:**
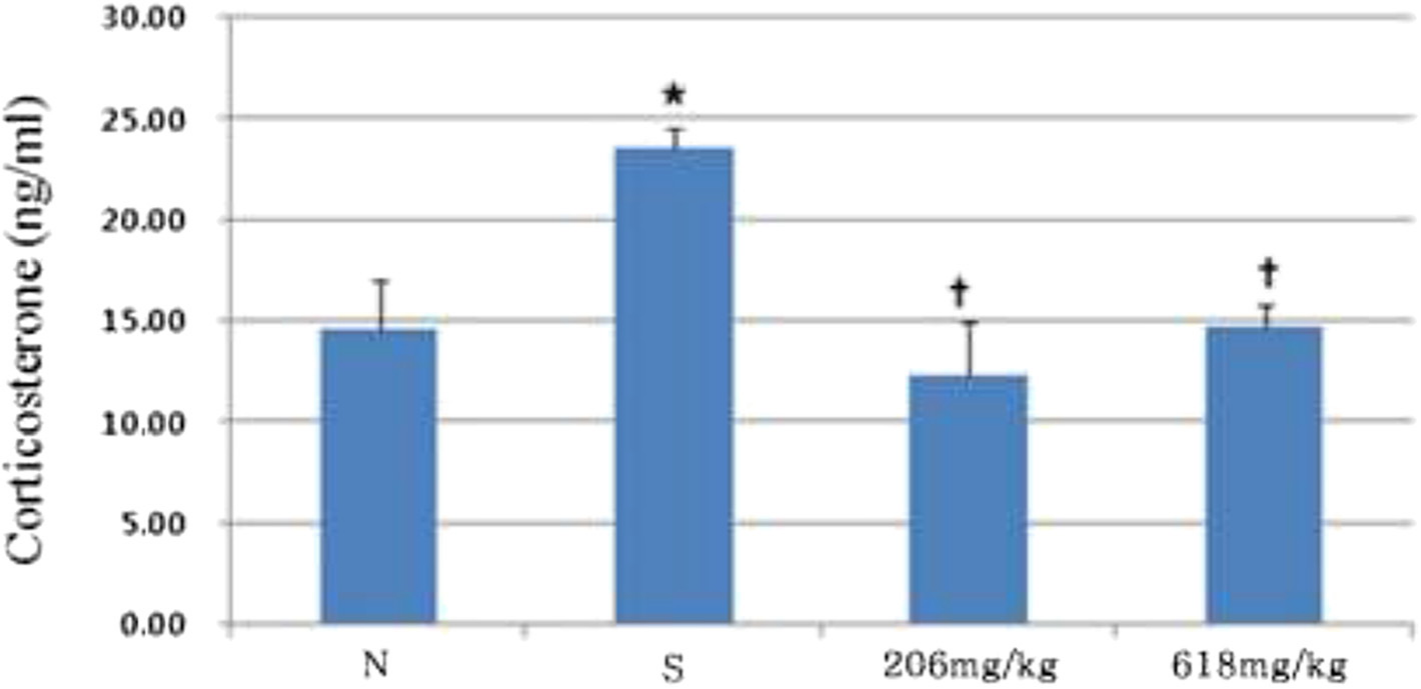
**Effects of exposure to chronic stress and herbal mixture on corticosterone levels.** Data are presented as means ± SEM of ten mice per group. ^*^ *P* < 0.05 vs. normal, ^†^ *P* < 0.05 vs. stress.

**Figure 7:**
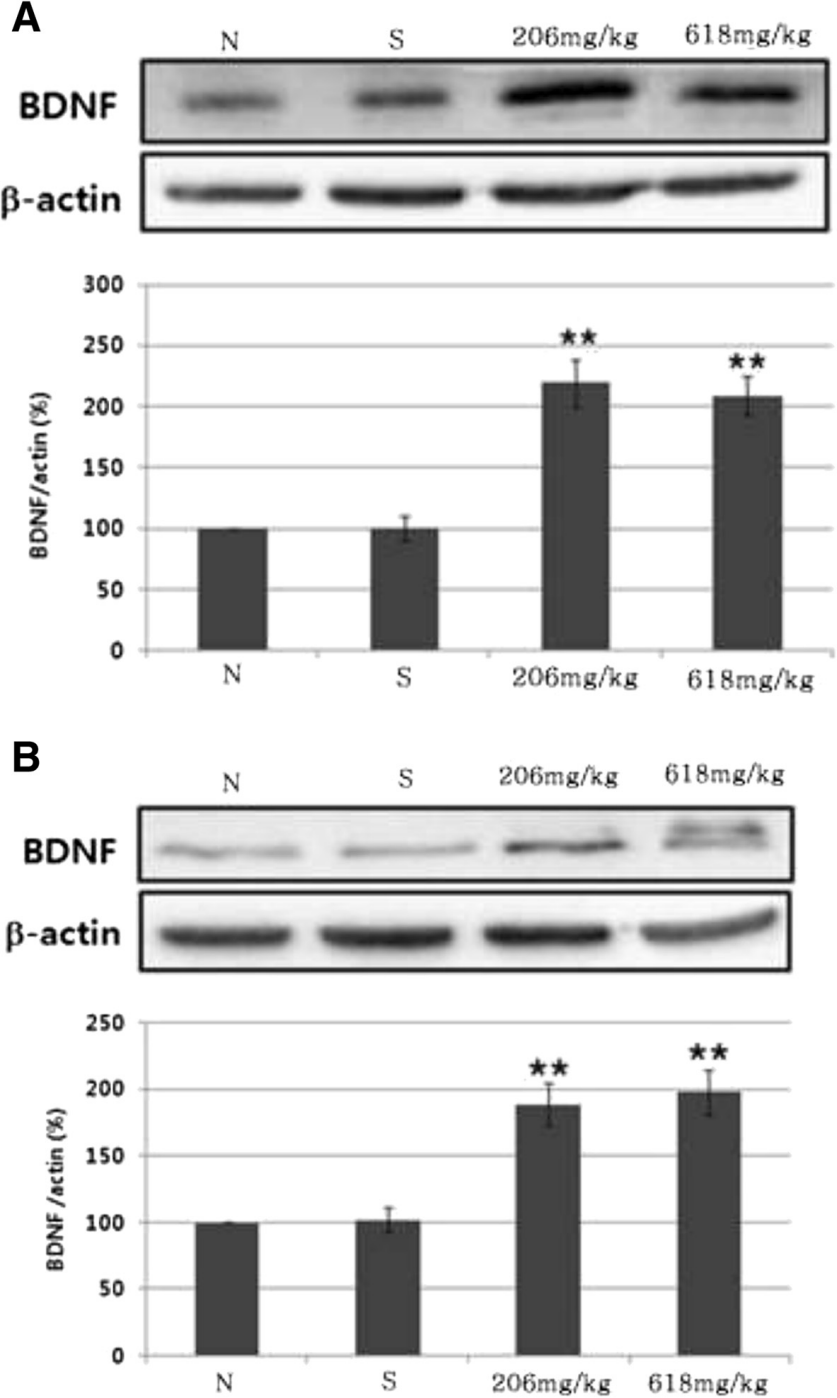
**Effects of the oral administration of herbal mixture on protein levels of BDNF in the hippocampi (A) and cortices (B).** Data are presented as means ± SEM of three determinations. ^**^ *P* < 0.01 vs. stress.

We examined whether herbal mixture attenuated depression in mice under stress conditions. We first performed the Y-maze test for spatial working memory. Only the stress mice displayed a reduced level of spontaneous alternation in the Y-maze test compared to that of the normal control group (p < 0.01, Figure 4A). However, mice in the experimental group, which were fed herbal mixture for 3 weeks, exhibited an increased level of spontaneous alternation (p < 0.01, Figure 4A). In addition, the total number of maze arm entries was not different among the groups (Figure 4B). We then examined the effects of herbal mixture on learning and memory using a passive avoidance test. Administration of low dose herbal mixture attenuated the learning memory impairment induced by stress (p < 0.05, Figure 4C). This finding indicates that herbal mixture improved the stress induced memory impairment under stress conditions. We further examined the effect of herbal mixture on the forced swim test. In 5 min swim test, normal mice were immobile for 229.91 ± 1.0 sec and stress mice were immobile for 237.20 ± 0.4 sec (p < 0.01, Figure 5A). The immobility was the sum of the durations of floating, twitching and kicking behaviors. We observed that herbal mixture administration did not induce any significant change in immobility when compared to the stress group. But it trended in favor of herbal mixture. When climbing and swimming behaviors were combined, stress mice were less active with 2.80 ± 0.4 sec than those in normal group with 10.09 ± 1.0 (p < 0.01, Figure 5B). Herbal mixture caused improvement of activity compared with stress group but as a non-significant trend. The effects on immobility and swimming observed after the administration of high dose of herbal mixture were similar to those observed after the administration of low dose of herbal mixture. On the other hand, herbal mixture produced highly significant reduction in the immobility time in the tail suspension test (p < 0.01, Figure 5C). Figure 6 illustrates the serum corticosterone concentration among groups. Mice in the herbal mixture groups demonstrated lower serum corticosterone concentration than those in the stress group (p < 0.05). As expected, mice exposed to the chronic stress paradigm had elevated corticosterone level compared to those in the normal group (p <0.05). Effects of the oral administration of herbal mixture on protein levels of BDNF in the hippocampi and cortices are shown in Figure 7. Statistical analysis indicated a significant effect of administration with herbal mixture. There were 54.55% (p < 0.01) and 52.15% (p < 0.01) increase in the levels of BDNF in the hippocampal tissues after administration with 206 and 618 mg/kg herbal mixture as compared with stress group, respectively. In the cortical tissues, 206 mg/kg herbal mixture significantly increased the BDNF level (46.03%, p < 0.01 vs. stress) similar to that of 618 mg/kg herbal mixture (48.49%, p < 0.01 vs. stress).

## Discussion

Numerous medicinal herbs have been employed as a form of mixture or decoction since their effects can be manifested after total and final reaction by their constituent compounds when administered to humans. The herbal mixture used in the present study consists of *E. longana*, *H. cordata* and *D. japonica* which demonstrated an inductive effect on the expression of BDNF, pCREB and pAkt. These herbs have been used as a therapeutic agent for cerebral disease especially in Korea in conjunction with Sasang Constitutional Medicine which takes a typological constitution approach to holistic medicine by balancing an individual’s psychological, social and physical aspects to achieve wellness and increased longevity.

The present study demonstrated that oral administration of herbal mixture in both doses (206 and 618 mg/kg) alleviated stress induced learning and memory deficits for mice by remaining in the well-lit side of a two compartment apparatus and not entering the dark where it received the electrical stimulus. Since mouse innately gravitates to darkness, the animal has to suppress this instinct through pairing the aversive stimulus (electrical stimulus) with the desired compartment (dark chamber). Animals that do not remember the aversive stimulus will cross over earlier than animals that remember. In addition, it was clear that the level of exploratory activities was not affected by the spatial working memory of mice. Herbal mixture treatment also decreased the immobility time in the tail suspension test. In order to understand the molecular mechanism of herbal mixture induced antidepressant effects in the brain, the present study investigated BDNF protein levels in hippocampus and cortex of mice under stress and compared values with herbal mixture fed mice under stress. Treatment with herbal mixture showed a significant increase in BDNF translation level.

Chronic stress may lead to psychotic symptoms including depression and suicide [18]. Studies have shown that the hippocampus is affected by stress. Depression invoked by stress has been associated with neuronal atrophy and decreased volume of hippocampus [19–21]. Several clinical reports have shown depression-induced deregulation of serum BDNF concentration. Depressed patients were characterized by low serum BDNF levels [6,22,23], implying an inverse relationship between serum BDNF levels and the severity of depression. Support for this comes from a number of studies demonstrating that treatment with antidepressants has been shown to increase BDNF levels in serum [6,23,24] and plasma [25]. It is speculated that lower BDNF levels may be caused by dysregulation of BDNF expression. This was evidenced with decreased BDNF mRNA and protein levels in post-mortem hippocampus and frontal cortex of suicide victims [26]. Recent reports further support the speculation that antidepressant treatment increased BDNF protein levels in serum [27] and in both prefrontal cortex and hippocampus [28]. In the current study, herbal mixture administration with low and high dose increased the BDNF protein levels in hippocampus and cortex compared to the stress group. Herbal mixture might have significantly increased the BDNF transcript levels as well although they were not evaluated in our study.

Several types of stressors have been known to disrupt BDNF expression. For example, single (one day) or repeated (7 days) immobilization for 2 hours per day markedly reduced BDNF mRNA levels in the dentate gyrus and hippocampus [29]. This was later confirmed by other investigators who used the same stress paradigm [30]. Other types of stressors such as footshock [31], early maternal deprivation [32], social defeat [33] and chronic unpredictable stress before pregnancy [34] were correlated with decreased BDNF expression. In our study using immobilization as a stressor, no significant changes in BDNF protein level were observed in hippocampus and cortex. It can be proposed that stress induced changes in BDNF protein level may be involved in other brain regions. The other hypothesis is that immobilization stress duration was too long enough to neutralize the downregulation of BDNF expression. Interestingly, brief immobilization stress can induce BDNF expression as part of a compensatory response to preserve hippocampal homeostasis to cope with new stress [35].

Our study provides further data that herbal mixture is able to modulate serum corticosterone level in mice under stress conditions. This effect could be either of peripheral origin through a direct action of herbal mixture on adrenal glands, or of central origin via the hypothalamic-pituitary-adrenal axis. The inverse relationship between memory performance and corticosterone level in stress conditions from our study confirms the past report that chronic stress in rodents has mostly impairing effects on memory [36]. Moreover, a long term immobilization stress has been shown to affect spatial memory [37] which is in accordance with our data of short term immobilization paradigm.

## Conclusions

In conclusion, our study showed that herbal mixture administration has antidepressant effects in mice. Each herb induced the expression of BDNF, pCREB and pAkt. The administration of herbal mixture significantly increased BDNF protein expression in mouse hippocampus and cortex.
